# Generation of anti-TLR2 intrabody mediating inhibition of macrophage surface TLR2 expression and TLR2-driven cell activation

**DOI:** 10.1186/1472-6750-10-31

**Published:** 2010-04-13

**Authors:** Carsten J Kirschning, Stefan Dreher, Björn Maaß, Sylvia Fichte, Jutta Schade, Mario Köster, Andreas Noack, Werner Lindenmaier, Hermann Wagner, Thomas Böldicke

**Affiliations:** 1 Institute of Medical Microbiology, University Duisburg-Essen, D-45122 Essen, Germany; 2 Institute of Medical Microbiology, Immunology and Hygiene, Technische Universität München, Troger Str. 30, D-81675 Munich, Germany; 3 Department of Gene Regulation and Differentiation, Helmholtz Centre for Infection Research, Inhoffenstr. 7, D-38124 Braunschweig, Germany

## Abstract

**Background:**

Toll-like receptor (TLR) 2 is a component of the innate immune system and senses specific pathogen associated molecular patterns (PAMPs) of both microbial and viral origin. Cell activation via TLR2 and other pattern recognition receptors (PRRs) contributes to sepsis pathology and chronic inflammation both relying on overamplification of an immune response. Intracellular antibodies expressed and retained inside the endoplasmatic reticulum (ER-intrabodies) are applied to block translocation of secreted and cell surface molecules from the ER to the cell surface resulting in functional inhibition of the target protein. Here we describe generation and application of a functional anti-TLR2 ER intrabody (αT2ib) which was generated from an antagonistic monoclonal antibody (mAb) towards human and murine TLR2 (T2.5) to inhibit the function of TLR2. αT2ib is a scFv fragment comprising the variable domain of the heavy chain and the variable domain of the light chain of mAb T2.5 linked together by a synthetic (Gly_4_Ser)_3 _amino acid sequence.

**Results:**

Coexpression of αT2ib and mouse TLR2 in HEK293 cells led to efficient retention and accumulation of TLR2 inside the ER compartment. Co-immunoprecipitation of human TLR2 with αT2ib indicated interaction of αT2ib with its cognate antigen within cells. αT2ib inhibited NF-κB driven reporter gene activation via TLR2 but not through TLR3, TLR4, or TLR9 if coexpressed in HEK293 cells. Co-transfection of human TLR2 with increasing amounts of the expression plasmid encoding αT2ib into HEK293 cells demonstrated high efficiency of the TLR2-αT2ib interaction. The αT2ib open reading frame was integrated into an adenoviral cosmid vector for production of recombinant adenovirus (AdV)-αT2ib. Transduction with AdVαT2ib specifically inhibited TLR2 surface expression of murine RAW264.7 and primary macrophages derived from bone marrow (BMM). Furthermore, TLR2 activation dependent TNFα mRNA accumulation, as well as TNFα translation and release by macrophages were largely abrogated upon transduction of αT2ib. αT2ib was expressed in BMM and splenocytes over 6 days upon systemic infection with AdVαT2ib. Systemic transduction applying AdVαT2ib rendered immune cells largely non-responsive to tripalmitoyl-peptide challenge. Our results show persistent paralysis of TLR2 activity and thus inhibition of immune activation.

**Conclusion:**

The generated anti-TLR2 scFv intrabody inhibits specifically and very efficiently TLR2 ligand-driven cell activation *in vitro *and *ex vivo*. This indicates a therapeutic potential of αT2ib in microbial or viral infections.

## Background

Among pattern recognition receptors (PRRs), toll-like receptors (TLRs) are prominent as cellular sensors of extracellularly encountered whole microbes or viruses, or pathogen associated molecular patterns (PAMPs) [1]. Infection of hosts is associated with release of immune-stimulatory PAMPs such as LPS upon Gram-negative bacterial infection or lipoproteins and DNA upon virtually any bacterial infection [2]. Systemic presence of PAMPs at high concentrations and subsequent overamplification of immune responses through TLRs is recognized as a major cause of sepsis for which high serum concentrations of pro-inflammatory mediators such as nitrogen monoxide and TNFα, as well as consequent hypotension and disseminated coagulation are characteristic. Sepsis frequently culminates upon multi-organ failure in fatal septic shock [3]. Chronic inflammation for which persisting infection is viewed as one major trigger might be maintained through PRR activation [4]. Targeting of PRRs such as CD14, TLR4, and TLR2 in models of acute infection within which deliberate short term antagonism is achieved by systemic application of neutralizing mAbs has been shown to effectively inhibit unwanted immune responses [5-7]. However, strategies aiming at sustainable PRR antagonism have not yet been brought forward.

As opposed to blockade of the ligand binding domain of PRRs, inhibition of intracellular translocation of TLRs from the endoplasmatic reticulum (ER) to the cell surface or endosomal compartment would require a cytoplasmic entry to inhibit TLR function and rely on an alternative strategy. Since intracellular antibodies (intrabodies) can be targeted to specific subcellular compartments upon fusion to specific signal peptides (nucleus, ER, mitochondria) or expressed inside the cytoplasm, they might be helpful for targeting PRRs. Inside the subcellular compartment the intrabody binds its cognate target [8-10]. Thereby intrabodies might supplement knock-down approaches such as antisense RNA or RNAi/siRNA/shRNA application potentially inducing off-target effects [11,12]. Major advantages of intrabodies include high specificity, substantial length of active half-life, opportunity of targeting protein domains or epitopes, as well as inhibition of post-translational modification. An intrinsic prerequisite of the intrabody technique is availability of a mAb against a specific target protein. If this need is met, cloning of the two DNA sequences encoding the variable domains of the specific mAb allows construction of a single chain Fv intrabody construct fused to specific signal peptides for targeting of specific subcellular compartments.

Intrabodies residing within the ER due to an ER retention peptide fused to the C-terminus of the respective intrabody, have been shown to efficiently block translocation of otherwise secreted or cell surface bound molecules to the cell surface [13]. ER intrabodies have been applied successfully for inactivation of oncogenic receptors as well as inhibition of virus envelope proteins and virus-receptor molecules expressed on the surface of host cells. Recent reports provide examples for blockade of translocation from the ER to the cell surface of specific antigens such as vascular endothelial growth factor receptors (VEGFR) 2 and Tie2, the human immunodeficiency virus (HIV) 1 coat protein gp120, as well as the HIV-1 coreceptor CC-chemokin-receptor (CCR) 5 [10,14-17]. In addition several receptors of the immune system have been knocked down with ER intrabodies (e.g. IL-1 receptor, MHC I, CD147 and VCAM, [18-21]).

Specifically, ER intrabodies carrying C-terminally the ER retention peptide KDEL or SEKDEL bind to ER retention defective (ERD) 2 receptor localized at the inner surface of the ER and at the same time recognize their specific antigen. The ER intrabody-target protein complex is recycled via the cis Golgi network apparatus to the ER [22]. The intrabody-antigen complex retained in the ER is then being degraded possibly inside the ER [23] or by a cytoplasmic proteosome-dependent [24] or proteosome-independent pathway [[25])]. Intrinsically, ER intrabodies are folded correctly due to interaction with molecular chaperones and the oxidizing environment of the ER, which favors intra-domain disulfide bond formation [26].

Here we describe the generation and characterisation of an anti-TLR2 scFv ER intrabody (αT2ib). αT2ib binds intracellularly and specifically to TLR2 as shown by co-immunoprecipitation as well as efficient retention and accumulation of TLR2 inside the ER upon overexpression of αT2ib. As a consequence, TLR2 surface expression and TLR2-specific activation of murine macrophages is inhibited *in vitro *and *ex vivo*. Our data indicate a therapeutic potential of αT2ib since it sustainably prevents TLR2-driven inflammatory immune responses.

## Results

### αT2ib expression and functionality upon transient overexpression in HEK293 cells

**Figure 1:**
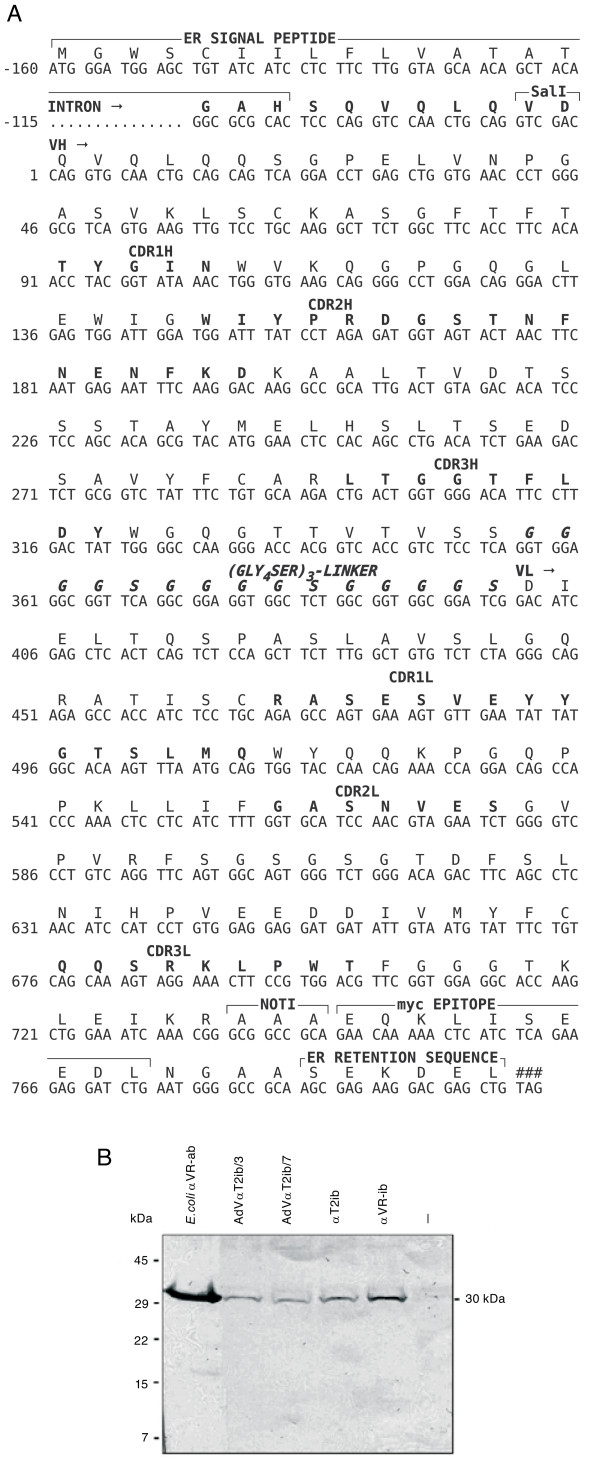
**Human/murine TLR2-cross-reactive intrabody assembly and expression**. *A*, Primary sequence of αT2ib. Shown are the coding (lower lane) and amino acid sequence (upper lane) of αT2ib including the ER signal peptide, the *myc *epitope and the ER retention sequence. The complementary-determining regions (CDR1-CDR3) of the variable domains of the heavy and light chain are printed in bold. The synthetic linker (shown in bold italic letters) localized between the VH and VL domains was introduced by assembly PCR (###, stop codon). *B*, Transient expression of the αT2ib in HEK293 cells. Comparative immunoblot analysis of αT2ib and AdVαT2ib cosmid clones 3 and 7 (/3,/7) expression in transiently transfected HEK293 cells. As positive controls, samples of *E. coli *periplasmic fraction of anti-VEGFR-2 scFv A7 (*E. coli *αVR-ab, left lane) and anti-VEGFR-2 intrabody scFv A7, αVR-ib (lane at the right side) transfected cells were applied. As negative control, HEK293 cells transfected with the vector pCMV/*myc*/ER without intrabody insert (right lane,-) were applied.

**Figure 2:**
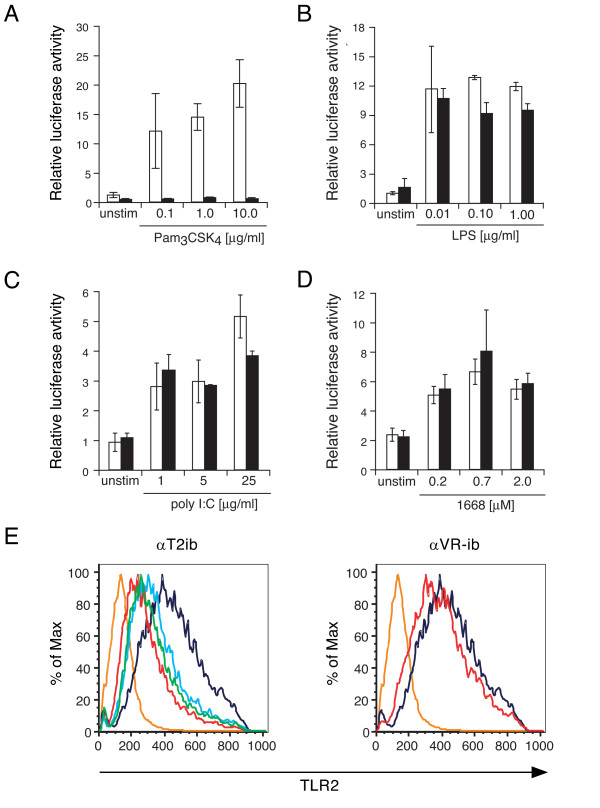
**Specificity of αT2ib function upon ectopic mouse TLR2 expression**. *A*-*D*, Assay of NF-κB driven reporter gene activation in HEK293 cells transiently coexpressing mTLR2 (*A*), mTLR4-MD-2 (*B*), hTLR3 (*C*) or mTLR9 (*D*) and αT2ib (black columns) or αVR-ib (white columns) and challenged as indicated (unstim, non challenged). (*E*) 3 × 10^4 ^human embryonic kidney (HEK) 293 cells per single well of a 6-well microtitre plate transfected with DNA plasmid driving expression of N-terminally-Flag-tagged human TLR2 were cotransfected with graded amounts of TLR2 specific intrabody (αT2ib) or control intrabody (αVR-ib) expression plasmid (line blue, 1 μg; green, 2 μg; red, 5 μg; line orange, 5 μg αT2ib expression plasmid, no primary antiserum; line purple, empty vector) to overexpress each of the two different intrabodies transiently. Upon 48 h cells from each well were subjected to flow cytometrical analysis using Flag-specific primary antiserum and PE coupled secondary antibody.

The sequence of the αT2ib expression construct was as illustrated (Fig. 1A). CMV promoter driven αT2ib expression in transfected HEK293 cells was detected by immunoblot (Fig. 1B) and immunofluorescence (data not shown). The apparent molecular weight of αT2ib was approximately 30 kDa (Fig. 1B). In order to analyse αT2ib function, NF-κB driven reporter gene activity in HEK293 cells overexpressing both mTLR2 and αT2ib upon TLR2 specific challenge was determined. Transfection of αT2ib expression plasmid DNA effectively inhibited TLR2 activity (Fig. 2A). In contrast, cellular activation through either TLR4-MD-2, TLR3, or TLR9 was not influenced by αT2ib coexpression (Fig. 2B-D). Another ER intrabody recognizing vascular endothelial growth factor receptor (VEGFR/KDR) 2 did not diminish TLR2 activation (Fig. 2A and data not shown). In addition, an αT2ib dose-dependent inhibition of TLR2 cell surface expression was observed (Fig. 2E). Moreover, the control-intrabody αVR-ib hardly diminished TLR2 cell surface expression to a detectable extent (Fig. 2E, right diagram).

### Colocalization of αT2ib and TLR2 inside the ER compartment and intracellular binding of αT2ib to TLR2

**Figure 3:**
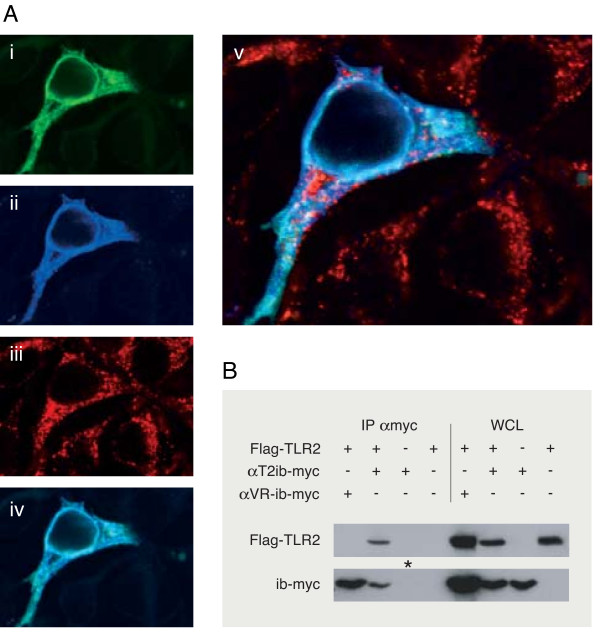
**Subcellular colocalisation and co-immunoprecipitation of αT2ib and mouse/human TLR2 upon transfection**. *A*, Immunofluorescence analysis by laser scanning confocal microscopy of fixed and permeabilized HEK 293 cells overexpressing mouse TLR2 and transiently transfected with αT2ib expression plasmid. i, Expression of αT2ib analysed using FITC labelled anti-*myc *antibody, ii, Expression of TLR2 visualized by polyclonal anti-mTLR2 serum and Cy5 labelled goat anti-rabbit antibody; iii, Expression of calnexin visualized by anti-calnexin antibody and Cy3 conjugated goat anti-mouse antibody, iv, Overlay of i and ii, v, Overlay of i, ii, iii. *B*, 3 × 10^5 ^HEK293 cells on a culture plate of 9 cm in diameter were transiently transfected with 5 μg expression plasmid DNA (to overexpress Flag-tagged human TLR2 and/or a *myc*-tagged intrabody (ib-*myc*) as indicated) plus 5 μg of empty vector where only one expression plasmid was used. Upon 48 h cells were incubated in 1 ml lysis buffer and either analysed by immunoblotting directly (WCL, 20 ml whole cell lysate) or upon immunoprecipitation (IP) using *myc *tag-specific antiserum (α*myc*) and protein A/G beads (except for *-tagged lane illustrating application of beads only).

Fluorescent labeling revealed colocalization of αT2ib with mTLR2 indicating complex formation of both molecules (Fig. 3A, iv). The αT2ib-mTLR2 complex resided within a lattice structure identical with the ER compartment. This interpretation was supported by costaining of the intrabody-TLR2 complex with the ER resident marker calnexin (Fig. 3A, v). Overexpression of αT2ib led to strong accumulation of TLR2 inside the ER. This accumulation was not seen when TLR2 was coexpressed with the unspecific control-intrabody (αVR-ib, data not shown). Specific intracellular binding of TLR2 and αT2ib was indicated further by co-immunoprecipitation (Fig. 3B, second lane on the left). Lack of immunoprecipitation of TLR2 with control intrabody (Fig. 3B, first lane on the left) in lysates of HEK292 cells expressing respective protein pairs demonstrated αT2ib specificity for its cognate antigen.

### Adenoviral αT2ib expression and effects in macrophages

**Figure 4:**
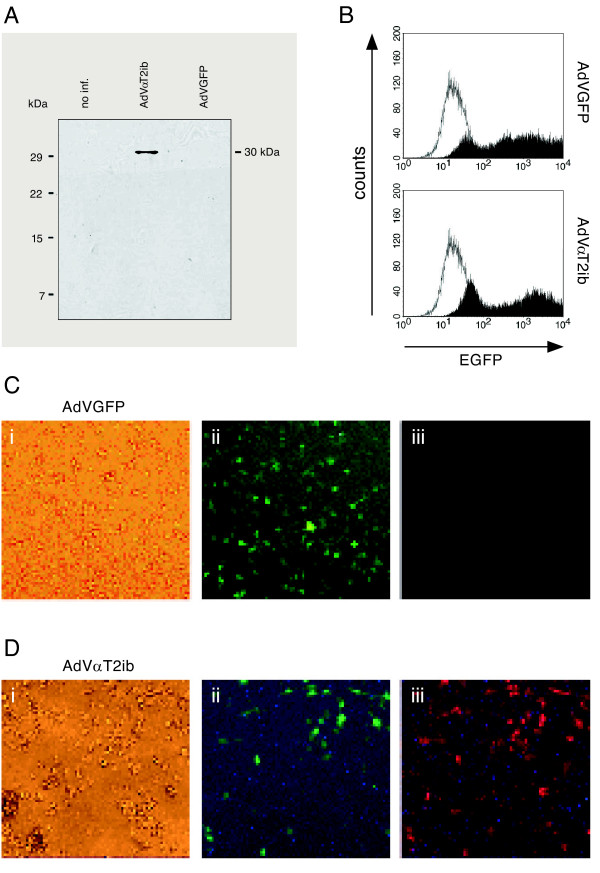
**Expression of adenovirally transduced αT2ib in RAW264.7 macrophages**. *A*, For *myc*-tag specific immunoblot analysis of RAW264.7 cell lysate the αT2ib protein band was visualized by application of a mouse anti-*myc *antibody. Non infected (no inf.) and AdVGFP infected cells were used as control. *B*, Transduction efficiency of RAW264.7 cells infected with AdVGFP or AdVαT2ib analysed by flow cytometry. Portion of infected cells was determined by analysis of green fluorescence by flow cytometry after 3 days of infection (empty area, noninfected cells; black area, cells infected as indicated). *C*, *D*, Immunofluorescence analysis by microscopy of permeabilized and fixed cells (i, Transmitted light; ii, Expression of EGFP visualized by UV-light; iii, Analysis of *myc *expression using a secondary TRITC labelled antibody).

**Figure 5:**
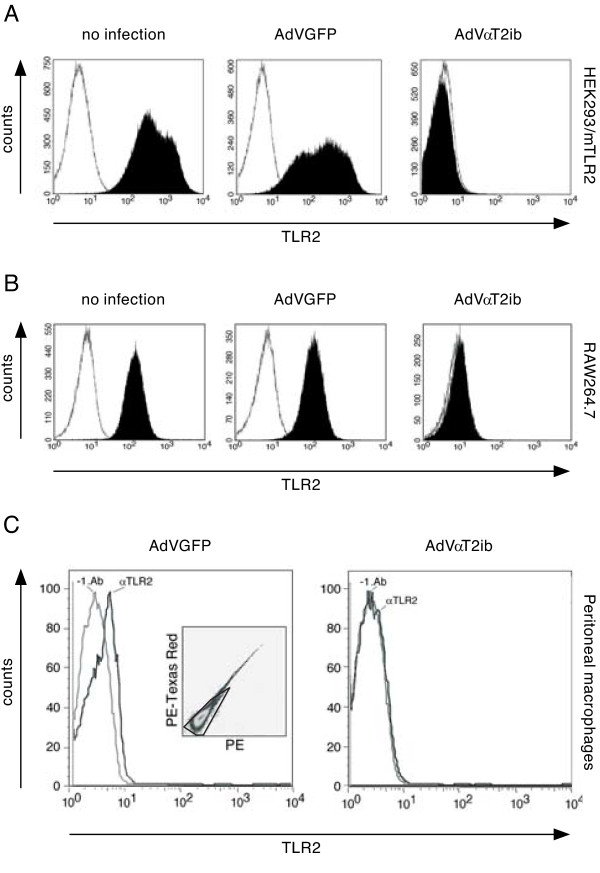
**Transduced αT2ib inhibits surface TLR2 expression by murine macrophages**. *A-C*, Flow cytometry of cell surface TLR2 of HEK293 cells overexpressing mouse TLR2 (*A*), RAW264.7 cells (*B*, Black area, PE staining correlating with TLR2 expression; white area, PE staining mediated by isotype control antibody; living infected cells were gated on GFP), and peritoneal macrophages (*C*) expressing αT2ib. Inset in left diagramm represents viability of primary macrophages (ethidium monoazide staining detected in PE-Texas Red versus PE channels, double positive cells excluded; grey line in main diagrams, no primary mAb, -1. Ab; black line in main diagrams, PE-labelled αTLR2 mAb T2.5).

**Figure 6:**
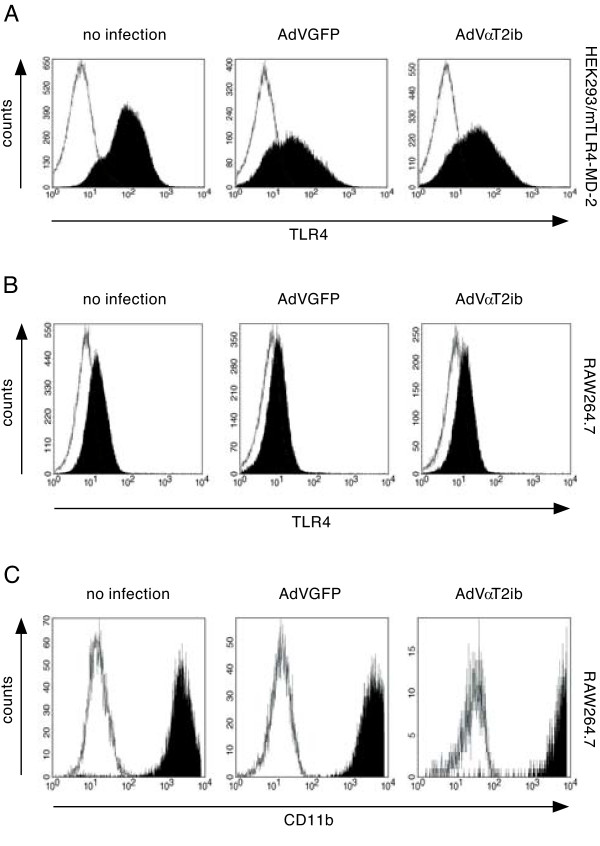
**Transduced αT2ib does not interfere with surface TLR4/CD11b expression**. *A-C*, Flow cytometry of TLR4 and CD11b cell surface expression of αT2ib expressing HEK293/mTLR4-MD-2 cells (*A*) and RAW264.7 cells (*B*, *C*, Black areas, specific staining; white areas, unspecific staining with isotype control mAbs). All viable infected GFP^+ ^cells were analysed.

To increase overexpression of αT2ib in macrophages and mice, we constructed an adenoviral vector and generated AdVαT2ib particles. Myc-tag specific immunoblot analysis of lysates of RAW264.7 macrophages that had been infected with AdVαT2ib or the control virus AdVGFP 3 days before revealed an apparent size of adenovirally transduced αT2ib of approximately 30 kDa (Fig. 4A). Adenovirus mediated αT2ib expression resembled that of αT2ib expressed upon DNA plasmid or DNA adenoviral cosmid transfection in respect to resultant protein size (compare Fig. 1B and 4A). Next, we analysed intrabody expression and bicistronically expressed EGFP by immunofluorescence determination through both, flow cytometry and microscopy. Both adenoviral constructs AdVGFP and AdVαT2ib transduced RAW264.7 macrophages equally effectively (Fig. 4B to 4D). Challenge of AdVGFP or AdVαT2ib infected RAW264.7 macrophages with TLR2 or TLR4 agonists for 4 h did not alter their EGFP-driven fluorescence (data not shown). Infection rates of RAW264.7 macrophages were in the 80% range as revealed by EGFP and myc-tag specific ELISA of cell lysates (Fig. 4B and data not shown). In order to analyse the effect of αT2ib expression on cell surface TLR2 expression, we performed flow cytometry of cells left uninfected and those formerly infected either with AdVαT2ib or AdVGFP. TLR2 cell surface expression was well detectable on non-infected HEK293 cells stable overexpressing murine TLR2, as well as on murine RAW264.7 macrophages (Fig. 5A and Fig 5B, left panels). Furthermore, adenovirally overexpressed EGFP had no substantial impact on cell surface TLR2 expression by HEK293/mTLR2 cells, RAW264.7 macrophages, as well as primary macrophages (Fig. 5A and 5B central pannel, Fig. 5C, left pannel). However, neither both of the two different cell lines, nor primary macrophages expressed surface TLR2 to a detectable degree given they had been infected with AdVαT2ib before (Fig. 5A to 5C, right pannels). Next we determined αT2ib specificity by comparative analysis of surface TLR4 expression. Both HEK293 cells overexpressing a Flag-tagged mTLR4-MD-2 complex as well as RAW264.7 macrophages displayed significant TLR4-specific staining (Fig. 6A and 6B, left pannels) which was reduced in HEK293 cells possibly due to reduced mTLR4-MD-2 complex expression in favor of expression of tranduced EGFP (Fig. 6A, middle pannel). Notably, TLR4 expression was not different to a significant extent on the cell surface of both cell lines given they were infected with either AdVGFP or AdVαT2ib before (Fig. 6A and 6B middle and right pannels) indicating specificity of αT2ib for TLR2. Furthermore, αT2ib did not interfere with expression of the macrophage marker CD11b on RAW264.7 cells (Fig. 6C).

### Inhibition of TLR2 specific signal transduction through αT2ib expression

**Figure 7:**
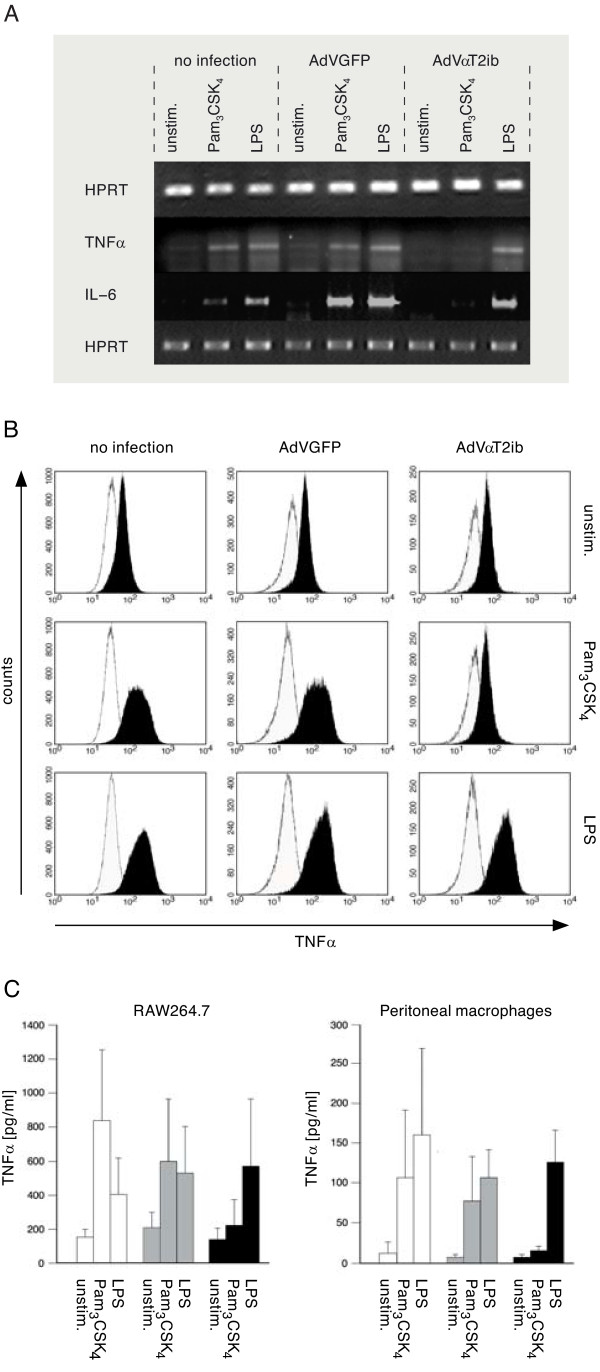
**Transduced αT2ib inhibits lipopeptide-but not LPS-induced macrophage activation**. TLR2 specific induction of cytokine mRNA accumulation and protein production. RAW264.7 macrophages were challenged with 100 ng/ml Pam_3_CSK_4 _or 100 ng/ml LPS for 4 h at room temperature. *A*, Cytokine mRNA accumulation in RAW264.7 macrophages was analysed upon RNA isolation and cDNA synthesis. Specific PCR amplificates were analysed semi-quantitatively (HPRT, hypoxanthine guanine phophoribosyl transferase). *B*, RAW264.7 macrophages were fixed/permeabilized and the TNFα release inhibited during stimulation. Intracellular cytokine was analysed by flow cytometry (black areas, specific staining; white areas, unspecific staining with isotype control mAb). All viable infected GFP^+ ^cells were analysed. *C*, Analysis of cytokine release to cell supernatant by ELISA (white columns, non infected; grey columns, AdVGFP infected; black columns, AdVαT2ib infected). Results of four independent experiments (p < 0.001).

Aiming at analysis of αT2ib effects on cell activation at the mRNA level we challenged RAW264.7 macrophages with TLR2 or TLR4 specific agonists upon specific adenoviral infection. Later, mRNA levels were determined by RT PCR. Within the time period of 4 h Pam_3_CSK_4 _and LPS challenge induced increase of cellular accumulation of both TNFα and IL-6 mRNA regardless of whether cells were left uninfected or had been infected with AdVGFP (Fig. 7A). Cells infected with AdVαT2ib responded similarly if challenged with LPS. In sharp contrast, neither IL-6 mRNA, nor TNFα mRNA were found in Pam_3_CSK_4 _challenged macrophages that expressed αT2ib upon adenoviral infection (Fig. 7A). Asking for TNFα induction on the protein level, we performed intracellular flow cytometry of intrabody expressing RAW264.7 cells upon gating on GFP positive cells. As shown in Fig. 7B, αT2ib expression inhibited intracellular TNFα accumulation whereas TLR4 specific activation induced through LPS challenge was not affected in respect to TNFα production. Adenoviral infection as such had no effect on constitutive or TLR ligand induced TNFα expression since AdVGFP infected cells produced as much of intracellular TNFα as non-infected macrophages (Fig. 7B). Thus, release of TNFα depended on TLR specific challenge. While noninfected cells responded to TLR2 or TLR4 specific challenge by releasing TNFα at substantial amounts which was true to an almost equal degree for cells infected with AdVGFP, cells that were infected with AdVαT2ib were unable to respond to a TLR2 specific challenge accordingly as shown for RAW264.7 and peritoneal macrophages (Fig. 7C).

### Effectiveness of systemic αT2ib transduction

**Figure 8:**
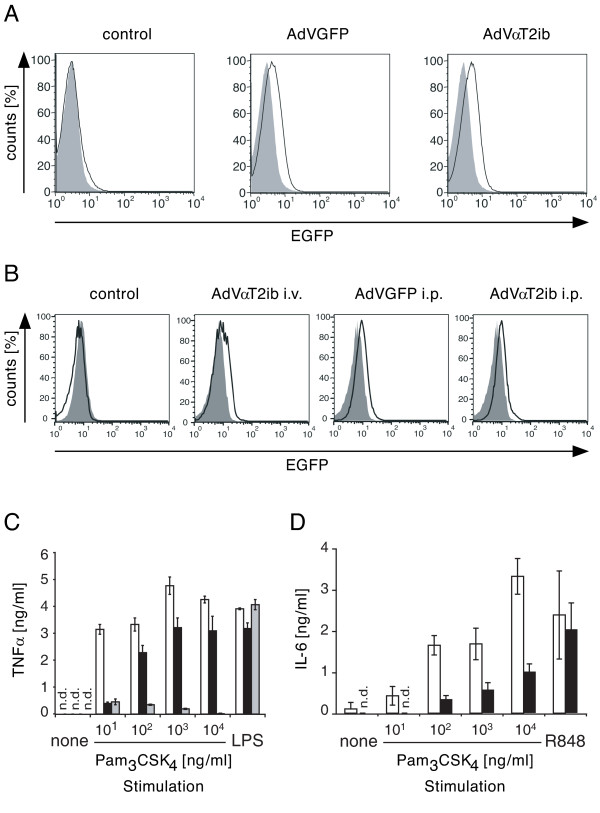
**Effective TLR2 inhibition through αT2ib-adenovirus infection *in vivo***. *A*-*D*, Mice were infected 6 d by intravenous (i. v.) or intraperitoneal (i. p.) injection (3 d after thioglycolate injection i. p. was performed) of 1 × 10^9 ^pfu of either AdVaT2ib or AdVGFP. *A*, Splenocytes were isolated from i. v. infected mice and stained with EMA and a CD11b specific antibody. Viable CD11b^+ ^splenocytes were analysed for EGFP expression by flow cytometry. As control splenocytes from non-infected mice were applied. *B*, Peritoneal wash out cells were drawn 6 d upon adenoviral infection as indicated or from non-infected mice (control). Cells that were adherent after 2 h of cell culturing were stained with EMA and analysed for EGFP expression. *C*, Peritoneal macrophages from mice infected with adenoviruses i. p. (white column, AdVGFP; black column, AdVaT2ib) or from a non-infected TLR2^-/- ^mouse (grey column) were challenged as indicated (none, non challenged) for 24 h after which supernatants were sampled for cytokine content by ELISA (one result of two similar results out of two independent experiments, n = 2 per group). *D*, Splenocytes from mice infected i. v. (white column, AdVGFP; black column, AdVaT2ib) were challenged for 24 h upon which supernatant cytokine concentration was determined by ELISA (representative result of one out of two independent experiments, n = 4 per group; n.d., not detected).

We proceeded with analysis of cells from systemically αT2ib transduced mice. Therefore, mice were adenovirally infected by i. v. injection of virus particles. 6 days after infection viable splenocytes were analysed for expression of bicistronically expressed EGFP by flow cytometry (Fig. 8A). Whereas splenocytes from non-infected mice were devoid of EGFP expression, mice infected with control virus or virus driving αT2ib expression expressed EGFP to well detectable degrees in their splenocytes (Fig. 8A). Next we analysed CD11b^+ ^peritoneal wash out cells from mice that had been adenovirally infected either i. v. or i. p. upon thioglycolate injection 3 days earlier for EGFP expression. While peritoneal macrophages from mice that had not been infected expressed no detectable EGFP, those of i. v. infected mice contained EGFP^+ ^cells. In contrast, in those mice that had been infected i. p. the entirety of the peritoneal macrophage population displayed increased EGFP expression as compared to cells from control mice (Fig. 8B, right panels). Consequently, peritoneal macrophages from mice formerly infected i. p. with AdVαT2ib but not from AdVGFP or non-infected mice were impaired in their ability to respond to low dose TLR2 agonist challenge while they were fully responsive to control stimulant (LPS, Fig. 8C). Similarly, splenocytes that descended from mice that had formerly been infected with AdVαT2ib but not from those infected with AdVGFP displayed diminished responsiveness to TLR2-specific but not to TLR7-specific challenge (Fig. 8D).

## Discussion

Means for prevention of excessive inflammatory immune actions are of substantial medical interest [2]. A major trigger of inflammation is infection, which is sensed through pattern recognition receptors (PRRs) by the host. Among signal transducing PRRs are TLRs, which as type I receptors mediate specific signals from the extracellular milieu into the cytoplasm [27]. Cellular PRRs such as cell surface expressed CD14, TLR4 or TLR2 have been blocked by application of soluble antagonists carried by the serum. However, antagonism of extracellular domains is only transient due to turn-over of surface receptors and limited half-time of serum contents. Treatment of long lasting or chronic inflammatory disease, however, might rely on sustained receptor blockage. Likewise, blockage of intracellularly localized PRRs would require an alternative approach. Referring to this, intrabodies have accomplished both persistent and intracellular blockade of receptors involved in cell cycle progression or cell growth [10]. Therefore, we generated an ER-bound intrabody towards TLR2 to evaluate its propensity for TLR2 blockade by TLR2 arrestment in the ER. αT2ib was derived from an antagonistic mAb towards both human and murine TLR2 [6] and inhibited translocation of TLR2 to the cell surface and TLR2 specific signal transduction both *in vitro *and *ex vivo*.

The variable domains of T2.5 were fused via a peptide linker and linked at the construct's C-terminus to the *myc *epitope followed by the ER retention peptide to a scFv-fragment named αT2ib (Fig. 1A). αT2ib expression was normal as compared to expression levels of previously generated intrabodies. Having an apparent size of 30 kDa, αT2ib specifically inhibited TLR2 driven cell activation since αT2ib effectiveness depended on TLR2 agonist dose and αT2ib did not inhibit activity of other TLRs such as TLR3, TLR4 or TLR9. Notably, αT2ib did not only inhibit cell surface expression and function of murine TLR2, but also of ectopically expressed human TLR2 (data not shown). Furthermore, a control intrabody towards VEGFR2 did not interfere with activity of any of the TLRs tested. αT2ib dose dependently inhibited TLR2 cell surface expression whereas the transfection of a control intrabody (αVR-ib) expression plasmid only marginally affected TLR2 cell surface expression.

αT2ib-driven inhibition was due to retention and accumulation of the formed intrabody-TLR2 complex inside the ER mediated by the ER retention sequence. We showed colocalisation of TLR2 and αT2ib inside the ER compartment upon synchronous staining with the ER-resident marker calnexin as well as specific binding of αT2ib to TLR2 by co-immunoprecipitation.

Aiming at effective transduction of murine macrophages we subcloned the αT2ib expression cassette into an adenoviral vector which drove bicistronic EGFP expression aside of αT2ib expression. Infection rates of RAW264.7 macrophages were in the 80% range as revealed by EGFP. Human macrophages specifically human alveolar macrophages have been reported to express low levels of CAR [28]. Nevertheless other authors used also adenoviral vectors to study biological effects in RAW 264.7 macrophages or peritoneal mouse macrophages [29,30]. Recently it was shown that the scavenger receptor A is responsible for uptake of adenovirus type 5 in J774 macrophages [31]. The exact mechanism of the entry of recombinant adenovirus in RAW 264.7 macrophages has not yet been studied. Since the RAW 264.7 macrophages also express the scavenger receptor A, entry of the adenovirus might be mediated by it. We suggest that it might be possible that the efficient transduction could also be due to macropinocytosis or phagocytosis triggered by the recombinant adenovirus [32].

The size of αT2ib expressed upon AdVαT2ib infection was similar as compared to αT2ib expression which was driven by transfected plasmid or cosmid. Notable, AdVαT2ib infection reduced surface TLR2 expression to almost baseline levels. Peritoneal macrophages infected *ex vivo *by adenovirus to express control protein expressed TLR2 at their surface to similar extent compared to non-infected macrophages. However, AdVαT2ib infection resulted in abrogation of TLR2 cell surface expression. Lack of interference of adenovirally transduced αT2ib with surface TLR4 expression emphasized TLR2 specificity (Fig. 6).

Inhibitory effects of αT2ib extended to TLR2 induced signal transduction since TLR2 specific induction of gene activities was ablated in addition to diminished surface TLR2 expression. In contrast, macrophages infected with AdVαT2ib did increase inflammatory cytokine mRNA accumulation upon LPS challenge (Fig. 7). At the same time house keeping gene expression was not impaired. Namely, both intracellular TNFα accumulation and cellular release of TNFα upon TLR2-specific challenge were at background level if αT2ib was expressed and in both cases TLR4 specific cellular challenge was unimpaired. These findings indicated both high effectiveness, as well as high specificity of TLR2 retention in the ER.

To translate results of our analyses *in vitro *to the systemic level, we infected naive mice with AdVαT2ib by i. v. injection, as well as mice that had been thioglycolate challenged 3 days earlier by i. p. injection of the adenovirus. 6 d after infection EGFP expression as an indication of αT2ib expression was evident upon infection i.v. in splenocytes regardless of whether mice had been infected with AdVαT2ib or AdVGFP. Even peritoneal washout cells expressed EGFP to a detectable degree regardless of whether mice had been transduced by infection through the i. v. or the i. p. route before. Consequently, those macrophages that originated from mice that had been infected with AdVαT2ib were refractory of responsiveness specifically to TLR2 specific challenge *in vitro *similar to cells from uninfected *TLR2*^-/- ^mice.

To our knowledge these data are first to show that intracellular arrestment of a TLR by an ER intrabody inhibits specific TLR activity. The specificity of mAbs might be an advantage outweighing potentially competing approaches such as application of agents that interfere with gene activation at the mRNA level [33]. Whether other vectors might accommodate the αT2ib encoding nucleic acid more appropriately remains an open question [34]. New approaches including transcriptional and transductional targeting are promising in this context [35,36]. Yet, our data clearly indicate highly effective function of αT2ib that extends from nearly abolished TLR2 cell surface expression to low cytokine release from cells upon confrontation with TLR2 agonists. Consequently, 6 d after initiation of systemic adenoviral transduction immune cells were resistant to TLR2 specific challenge. Further analysis will determine the time frame of αT2ib activity from adenoviral infection on. Intrabody mediated PRR blockade as compared to regular mAb mediated PRR blockage might not only constitute an exclusive way to target intracellularly localized receptors using mAbs but also be advantageous for blockade of those PRRs a possibly minor portion of which is expressed superficially such as TLR2. An obvious field of anti-TLR intrabody application might be chronic inflammatory diseases such as inflammatory bowel disease, asthma, or arthritis because it might call for sustained anti-inflammatory action. TLRs have been implicated as initial signal transmitters under such clinical conditions [2,4,37]. A future perspective is the evaluation of the efficacy of αT2ib in mouse models of chronic infections. Furthermore the intrabody strategy might be used to block TLR2 specific TNFα secretion of myeloid cells induced by factors secreted from tumour cells promoting metastasis [38].

## Conclusion

We generated and characterised an anti-TLR2 ER intrabody that inhibits specifically and very efficiently macrophage surface TLR2 expression and TLR2-driven cell activation *in vitro *and *ex vivo*. This indicates a therapeutic potential of αT2ib for treatment of TLR2-driven chronic inflammatory disease.

## Methods

### 1. Cells and mice

Murine RAW264.7 macrophages were obtained from the German strain collection (DSMZ, Braunschweig, Germany), Human embryonic kidney (HEK293)/murine (m)TLR2 and HEK293/mTLR4-MD-2 lines were established by stable transfection of mTLR2 and mMD-2 expression plasmids obtained from H. Heine (Research Center Borstel, Germany), D. Golenbock (University of Massachusetts Medical School, USA) and K. Myake (Institute of Medical Science, Tokyo, Japan). The mTLR4 coding sequence was reamplified from a RAW264.7 cDNA library. Peritoneal and bone marrow derived primary murine macrophages, as well as splenocytes were prepared as described and cultured for analysis without or after challenge with TLR ligands [39]. Macrophages and splenocytes were cultured in 50 μM Mercaptoethanol, 10% heat inactivated FCS, pen/strep as antibiotics at standard concentration in an incubator under regular cell culture conditions.

Adult matched C57BL/6 wild-type and TLR2^-/- ^mice [40] were used for experiments. The mice experiments followed internationally recognized guidelines and were approved by the Government of Upper Bavaria.

### 2. αT2ib assembly

Total RNA of the hybridoma T2.5 was isolated using the RNeasy Mini Kit (Qiagen; Hilden, Germany) and the cDNA synthesized using a "First-strand cDNA synthesis kit" (GE Healthcare, England). DNA fragments encoding the variable domains of the heavy and the light chain of T2.5 were amplified using consensus primer [41], for the variable domain of the heavy chain VH1Back-1: 5'-CAG GTS MAR CTG CAG SAG TCW GG (S = G or C, M = A or C, R = A or G, W = A or T) and VH1FOR-2: 5'-TGA GGA GAC GGT GAC CGT GGT CCC TTG GCC CC. The variable domain of the light chain was amplified applying VK2Back: 5'-GAC ATT GAG CTC ACC CAG TCT CCA and MJK1FONX: 5'-CCG TTT GAT TTC CAG CTT GGT GCC. The PCR products of the heavy chain and light chain were purified from an 1% agarose gel and directly used for assembly of the variable domain of the heavy and light chain with a linker sequence that creates a 15 amino acid sequence (Gly_4_Ser)_3 _between both domains. The linker was generated from the scFv A7 isolated by phage display [42] by a PCR with the primer LINK BACK: 5'-GGC ACC ACG GTC ACC GTC TCC TCA and LINK FOR 5'-TGG AGA CTG AGT GAG CTC GAT GTC. The assembly PCR was performed in two steps. In the first reaction equimolar amounts of the DNA of the VH and VL domain and the synthetic linker were incubated with 25 pmol of the primer VH1 Back and MJK1FONX in a PCR of 50 μl containing 1.0 mM dNTPs, 2.5 mM MgCl_2 _(Qiagen), 5 μl 10 × PCR buffer (Qiagen) and 1 μl (1 U) *Taq *Polymerase (Qiagen). PCR amplification was performed by incubation for 20 cycles at 94°C for 1.5 min and 65°C for 3 min.

In a second step 25 pmol of the VH1Back primer containing a *Sal *I restriction site and 25 pmol of the MJK1FONX primer containing a *Not *I restriction site was added and the volume filled up with dNTPs (0.4 mM), 5 μl 10 × PCR buffer, H_2_O and 1 μl *Taq *Polymerase (1 U) to 100 μl. Incubation was performed for 30 cycles at 94°C for 1 min, 55°C for 2 min and 72°C for 2 min. This results in a complete scFv intrabody gene.

After purification of the assembled scFv from a 1% agarose gel the PCR product was cloned into linearised pCR2.1 vector containing 3' T overhangs (Invitrogen, Karlsruhe, Germany). The ligated DNA was transformed into *E. coli *Top 10 and after blue-white screening, positive clones were restricted with *Sal *I and *Not *I and respective inserts ligated into *Sal *I and *Not *I cleavage sites upon dephosphorylation with Shrimp alkaline phosphatase (USB Corporation, Cleveland, USA) of pCMV/*myc*/ER vector (Invitrogen). This vector encodes an ER signal peptide, contains a multicloning site (*Pst *I, *Sal *I, *Xho *I, *Not *I) as well as encodes a *myc *epitope and the ER retention signal SEKDEL. After cloning sequences encoding both a *myc *tag and an ER retention motif were fused 3'-terminally to the preceding anti-TLR2 scFv fragment. This plasmid drove expression of the scFv-*myc*-SEKDEL ER retention signal construct, which was identical to αT2ib.

### 3. Construction of an adenoviral vector for αT2ib transduction, production of recombinant virus, and infection *in vitro *and *in vivo*

Constructing an adenoviral vector carrying a bicystronic expression cassette driving expression of ER intrabody αT2ib and the reporter gene EGFP a strategy comprising two cloning steps was pursued. In the first step the intrabody expression cassette was cloned into the vector pGEM/IRES/EGFP [43] containing the IRES sequence of the poliovirus followed by the reporter gene EGFP. In a second step the bicystronic expression cassette containing the CMV promotor, the αT2ib coding sequence, the IRES sequence and the reporter gene EGFP was ligated into the adenoviral cosmid vector pAdcos45EGFPC1 [43].

First the plasmid pCMV/*myc/*ER containing the anti-TLR2 ER intrabody gene (αT2ib) coding sequence was linearised using *Eco *RI. To generate a *Sfi *I restriction site the linearised vector was ligated with the oligonucleotid LINKER ADENO: 5'-AAT TGC **GGC CGC CAT GGC C**GC (*Sfi *I restriction site is marked bold). 5 μg of the linker containing the *Sfi *I restriction site was ligated with 1 μg of the linearised vector using 4.5 Units T4 DNA Ligase (Promega, Germany) overnight at 20°C in a volume of 15 μl. Excess of the fragment was removed by dialysis of the sample against 10 mM Tris-HCL, 0.1 mM EDTA and the ligated DNA was transformed into *E. coli *DH5α. From a positive clone (pCMV/*myc*/ER/αT2ib/*Sfi *I) the αT2ib gene with the CMV promoter was cut out by restriction with *Sfi *I and *Xba *I and ligated into dephosphorylated pGEM/IRES/EGFP restricted with *Sfi *I and *Avr *II. After transformation into *E. coli *DH5α positive clones (pGEM/αT2ib/IRES/EGFP/*Sfi *I) containing the bycistronic expression cassette of the αT2ib gene and the reporter gene EGFP were used for the second step. pGEM/αT2ib/IRES/EGFP/*Sfi *I was restricted with *Pvu *II and *Xba *I and the bicystronic expression cassette encompassing the CMV promotor was ligated into the adenoviral vector pAdcos45 EGFPC1 from which the EGFP gene with the CMV promoter was removed by restriction with *Swa *I and *Xba *I. The adenoviral vector pAdcos45 EGFPC1 contains the genome of a replication deficient adenovirus type 5 subgenus C in which the E1 and E3 region is deleted [43]. The adenoviral vector contains the reporter gene EGFP. Its expression is driven by the immediate early CMV promoter.

After ligation the adenoviral cosmid DNA was packaged *in vitro *and transduced into *E. coli*. Two independent cosmid clones, namely AdVαT2ib/3 and AdVαT2ib/7 comprising identical sequences, were used for the generation of recombinant adenovirus. For production of AdVαT2ib particles HEK293 cells were transfected with 10 μg cosmid DNA in 80cm^2^cell culture flasks grown to 60% - 80% confluence. After 7-14 days viral plaques were visible. The recombinant adenovirus was propagated as described [44] and purified by CsCl gradient ultracentrifugation. For infection with the recombinant adenovirus RAW264.7 and primary peritoneal macrophages were cultured until 80% confluence was reached. Infection was carried out at a multiplicity of infection (moi) of 10 for the control virus AdVGFP which overexpressed EGFP but not an additional protein and at a moi of 100 for AdVαT2ib with infection buffer (PBS containing 2% FCS) for 1 h at room temperature since it resulted in equal EGFP expression levels. After infection MEM containing 5% FCS was added and the cells were incubated for 1 day to 3 days after which expression of the reporter EGFP was analysed by measurement of immunofluorescence and flow cytometry. Mice were infected by intravenous (i. v.) or intraperitoneal (i. p.) injection of 1 × 10^9 ^plaque forming units (pfu) of AdVαT2ib or control virus (AdVGFP) and analysed 6 days thereafter upon removal of cells by assaying *ex vivo/invitro *using ELISA.

### 4. Immunoblot and immunofluorescence analysis

Transiently intrabody expression plasmid transfected HEK293 cells and with recombinant adenovirus transduced RAW264.7 macrophages were lysed by incubation in lysis buffer (150 mM NaCl, 20 mM Tris/HCl pH7.4, 1% Triton-X-100, 10 mM EDTA, 100 μM vanadate, 1% Trasylol, 1 mM PMSF, 1 mM zinc acetate) for 20 min on ice. Lysates were centrifuged for 15 min in a table top centrifuge and a supernatant aliquot representing 5 × 10^5 ^cells was loaded on a lane of a PAA gel (12.5%) for subsequent SDS-PAGE. After blotting the membrane was blocked in 2.5% skimmed milk in PBS containing 0.05% Tween 20 at room temperature for 1 h. The intrabody band was visualized by using a mouse anti-myc antibody (Santa Cruz Biotechnology, Heidelberg, Germany) and a goat anti-mouse IgG Fcγ antibody coupled with horseradish peroxidase (Dianova, Hamburg, Germany). The blot was developed using 3,3'-diaminobenzidine tetrahydrochloride (DAB) liquid substrate (Sigma, Deisenhofen, Germany). For immunofluorescence based expression assay by microscopy or FACS the reporter gene product EGFP was visualized using UV light and a FITC filter. For analysis of intrabody expression cells were fixed with 3.7% formaldehyde (15 min at room temperature), washed 3 times with PBS and further permeabilized with 0.1% Triton X-100 for 10 min. After washing with PBS for 5 times, cells were blocked with PBS containing 3% BSA for 1 h at room temperature. Antibody incubation was performed with mouse anti-*myc *antibody and a TRITC labeled goat anti-mouse IgG antibody (Dianova).

### 5. NF-κB dependent luciferase assay in HEK293 cells overexpressing both specific TLRs and αT2ib

3 × 10^4 ^human embryonic kidney (HEK293) fibroblastoid cells were seeded per well of a 96-well cell culture plate and transfected with plasmids directing constitutive expression of mTLR2 or other TLRs, αT2ib or control intrabody (anti-VEGFR 2 intrabody scFv A7, αVR-ib [45], and Renilla luciferase, as well as NF-κB dependent expression of firefly luciferase. After 24 h cells were challenged with TLR agonists *E. coli *O111:B4 LPS (Sigma), tripalmitoylated hexapeptide (Pam_3_CSK_4_, EMC microcollections), poly I:C (Sigma), or oligodeoxynucleotide (1668, TIB Molbiol) for additional 16 h and lysed subsequently for analysis of luciferase activity.

### 6. Subcellular colocalisation and co-immunoprecipitation of αT2ib and mouse/human TLR2

For colocalisation HEK293 cells overexpressing mouse TLR2 were grown on sterile coverslips and transiently transfected with αT2ib expression plasmid. The cells were washed once with PBS-0.05% Tween 20, fixed for 10 minutes with 4% formaldehyd followed by permeabilisation of the cells with 0.1% Triton X-100 for 10 min at room temperature. After blocking with 3% BSA triple staining of αT2ib, mouse TLR2 and Calnexin was performed with polyclonal anti-mouse TLR2 serum [46], anti-calnexin antibody (Abcam, Cambridge, UK, clone AF18) and subsequent incubation with a FITC labelled goat anti-myc antibody, Cy3 labelled goat anti-mouse antibody (Dianova) and Cy5 labelled goat anti-rabbit antibody (Dianova). Incubation of antibodies was performed over a period of 1 h at room temperatur. Between incubation steps cells were washed 3 times with PBS-0.05% Tween 20. The coverslips were embedded in Moviol (Merk, Darmstadt, Germany) and analysed with a laser scanning confocal microscope (LSM 510 META, Carl Zeiss). Co-immunoprecipitation was performed as described before [46]. Briefly, each partner of protein pairs to be analysed for their potential to interact in a cellular context were overexpressed as fusion proteins in HEK 293 cells upon transfection by Ca_3_(PO_4_)_2_-DNA precipitation. Specifically, human TLR2 was coupled to an N-terminal Flag-tag while intrabody constructs contained C-terminal myc-tags (see Fig. 1a exemplarily). Subsequently, cells were lysed (0.5% NP40, 150 mM NaCl and further ingredients [46]) upon which nuclei were removed by centrifugation. Myc-specific antibody (Sigma) and protein G beads (Santa Cruz) were added synchronously and lysates incubated on a roller at 4°C for 16 h. Upon 5 washes with lysis buffer sample buffer was added and samples were subjected to SDS-PAGE and analysed upon blotting using tag-specific antibodies (Sigma).

### 7. Analysis of TLR2 and TLR4 cell surface expression of transfected HEK293 cells and macrophages

αT2ib expression plasmid transfected HEK293 cells overexpressing mTLR2, as well as AdVαT2ib infected RAW264.7 and primary macrophages derived from bone marrow were stained with murine/human TLR2 specific T2.5 or mTLR4 specific UT41 to determine surface expression of specific TLRs by flow cytometry. Staining with antibodies was performed for 30 min at 4°C in a 96-well microtitre plate (Nunclon™ Surface plate, Nunc) in 100 μl PBS containing 2% FCS (Invitrogen) using a phycoerythrin-labelled anti-mouse TLR2 antibody (clone T2.5, HBT) or a phycoerythrin-labelled anti-mTLR4 antibody (clone UT41, HBT). As isotype control a phycoerythrin labelled mouse IgG1κ (clone P3, HBT) was used. Cells were washed once with PBS containing 2% FCS and resuspended in 300 μl PBS containing 2% FCS and 10 μg/ml propidiumiodide for subsequent analysis using a FACS Calibur™ (Becton Dickinson).

### 8. Analysis of intracellular TNFα by flow cytometry

2 × 10^6 ^RAW267.4 macrophages in one well of a 6-well microtitre plate (uninfected, infected with AdVGFP or AdVαT2ib) in 1 ml medium were stimulated with 100 ng/ml tripalmitoylated hexapeptide Pam_3_CSK_4 _(EMC microcollections) or 100 ng/ml LPS (Alexis) for 4 h at room temperature. Fixation, permeabilization, and intracellular TNFα transport inhibition were performed using cytofix/cytoperm™ plus fixation/permeabilization and golgiplug™ protein transport inhibitor (BD Biosciences Pharmingen). Challenge with TLR agonists was performed in the presence of 1 μl golgi stop ™ solution in 1 ml medium. Cells were washed once with PBS containing 2% FCS and resuspended in 250 μl fixation/permeabilization solution and incubated for 20 min at 4°C. After washing the pellet once with Perm/Wash™ buffer Fcγ-receptors were blocked by incubation with anti-CD16/CD32 antibodies (BD Bioscience Pharmingen) for 30 min at 4°C. The cells were washed and stained intracellularly as described above for cell surface staining. Antibodies used were a phycoerythrin-labelled hamster anti-mouse/rat TNFα antibody (clone TN3-19.12) and isotype control phycoerythrin-labelled hamster IgG_1 _antibody (clone G235-2356, both BD Biosciences, Pharmingen).

### 9. Analysis of TNFα and IL-6 mRNA accumulation by reverse transcription (RT) and subsequent PCR mediated amplification

After cellular challenge with 100 ng/ml Pam_3_CSK_4 _or 100 ng/ml LPS for 4 h cellular RNA was isolated (RNeasy, Qiagen) and cDNA synthesized using 1 μg RNA and a random hexamer primer according to supplier instructions (GE Healthcare). Amplification of murine TNFα and IL-6 mRNA was performed by PCR using the following primers: TNFα reverse: 5'-ATGAGCACAGAAAGCATGATC and TNFα forward: 5'-CACAGAGCAATGACTCCAAAG, as well as IL-6 reverse: 5'-ATGAAGTTCCTCTCTGCAAGA and IL-6 forward: 5'-GGTTTGCCGAGTAGATCTCAA. The PCR was carried out in 20 μl of PCR buffer (1 mMTris-Cl, 10 mM KCL, 2 mM (NH_4_)_2_SO_4_, 35 mM MgCl_2_, pH 8.0), 2.5 mM dNTPs, 0.5 U of *Taq *DNA polymerase (Qiagen) and 10 pmol primers (Operon). Hypoxanthine-guanine phosphoribosyl transferase (HPRT) mRNA was amplified as control (HPRT reverse: 5'-TCAACGGGGGACATAAAA, HPRT forward: 5'-ATTCAACTTGCGCTCATCTT).

### 10. ELISA

TNFα and IL-6 concentrations in supernatants were analysed by application of ELISA kits according to supplier instructions (BD Biosciences). Aside of other TLR ligands (see above), a TLR7 specific compound (R848, Alexis) was applied to primary cells.

## Abbreviations

RAW 264.7: Mouse leukaemic monocyte macrophage cell line; scFv: single-chain variable region fragment; αVR-ib: ER intrabody against vascular endothelial growth factor receptor (VEGFR) 2; MD-2: coreceptor of TLR4; Pam_3_CSK_4_: triacylated synthetic lipoprotein (TLR2 ligand); LPS: lipopolysaccharide (TLR4 ligand); EGFP: enhanced GFP; WCL: whole cell lysate; Poly I:C (polyinosinic:polycytidylic acid (ligand of TLR3); ODN 1668: synthetic oligodeoxynucleotide (ligand of mouse TLR9); EMA: epithelial membrane antigen; R848/Resiquimod (Ligand of TLR7/TLR8).

## Author's contributions

CK designed the study, performed experiments and wrote the manuscript. SD cotransfected TLR2 and αT2ib for functional assaying and co-immunoprecipitaion. BM carried out flow cytometry analysis, RT PCR and intracellular FACS. SF carried out NF-κB/luciferase assays, ELISAs, and FACS analyses. JS constructed the recombinant adenovirus and documented expression of αT2ib. MK analyzed subcellular colocalisation of αT2ib and TLR2. AN carried out the ELISA experiments with RAW 264.7 and peritoneal macrophages. WL drafted the experiments performed with the recombinant adenovirus and supported us producing the recombinant intrabody adenovirus and controlvirus. HW has designed the study and provided antibodies and mice and has given final approval of the version to be published. TB conceived, drafted and designed the study, wrote the manuscript and analyzed colocalisation of αT2ib and TLR2 inside the ER. All authors read and approved the final manuscript.
